# Preparing of Interdigitated Microelectrode Arrays for AC Electrokinetic Devices Using Inkjet Printing of Silver Nanoparticles Ink

**DOI:** 10.3390/mi8040106

**Published:** 2017-04-01

**Authors:** Van-Thai Tran, Yuefan Wei, Wei Jing Liau, Hongyi Yang, Hejun Du

**Affiliations:** 1 Singapore Centre for 3D Printing, School of Mechanical and Aerospace Engineering, Nanyang Technological University, 50 Nanyang Avenue, Singapore 639798, Singapore; thaitran001@e.ntu.edu.sg; 2 School of Mechanical and Aerospace Engineering, Nanyang Technological University, 50 Nanyang Avenue, Singapore 639798, Singapore; WeiYF@ntu.edu.sg (Y.W.); LIAU0024@e.ntu.edu.sg (W.J.L.); 3 Singapore Institute of Manufacturing Technology, 71 Nanyang Drive, Singapore 638075, Singapore; mybhy@hotmail.com

**Keywords:** additive manufacturing, inkjet printing, silver nanoparticles, alternating current (AC) electrokinetics, dielectrophoresis, AC electro-osmosis

## Abstract

The surge in popularity of lab-on-chip applications has set a new challenge for the fabrication of prototyping devices, such as electrokinetic devices. In such devices, a micro-electrode is the key component. Currently, microelectromechanical systems (MEMS) processes such as lift-off and etching techniques are employed to prepare the micro-sized conductive patterns. These processes are time-consuming, require a material removal step, clean-room facilities, and the utilisation of harmful chemicals. On the other hand, rapid fabrication is required by researchers designing such devices to test their functionality. Additive manufacturing technology such as the inkjet printing of conductive material is one potential solution to achieve that objective. In this study, we report the utilisation of inkjet printing for the rapid prototyping of alternating current (AC) electrokinetic devices on a rigid glass substrate. The non-lithographical and vacuum-free process for the fabrication of a microfluidic device was demonstrated. The smallest feature size of 60 μm was successfully printed. The crystalline structure of the printed material under different curing temperatures was characterised. It was found that these treatment conditions affect electrical conductivity. Although a low-temperature sintering process was applied, low resistivity was obtained. An AC electrokinetics device for the manipulation of microparticles has been prepared to illustrate such printed silver micro-patterns. The results strongly support the idea that inkjet printing is a powerful and cost-effective prototyping tool for researchers who work with electrokinetic devices.

## 1. Introduction

Research in additive manufacturing—also known as 3D printing—has been booming in recent years. Additive manufacturing uses processes that gradually add material to build an object from a digital design [1]. Hence, it is capable of constructing complicated structures that are difficult to achieve using conventional subtractive methods [2]. Aside from the printing of structural material, the demand for the rapid preparation of functional devices has been raised in order to utilise the advantages of 3D printing [3,4,5].

Meanwhile, lab-on-chip systems are important tools for small-size biological sampling, where the analysis can be performed with a tiny volume of fluid. These devices can handle microscopic particles such as cells, bacteria, and DNA [6]. Advanced applications of lab-on-chip devices include water pollution monitoring [7], drug delivery [8], and device fabrication [9].

Alternating current (AC) electrokinetics is a set of techniques to manipulate small-sized particles with the effect of a non-uniform electric field. AC electro-osmosis is an AC electrokinetic technique that creates fluid motion with the migration of ions under the effect of tangential electric fields on the electrode surface [10]. The induced hydrodynamic drag force causes the movement of suspended particles [11]. Dielectrophoresis (DEP) is another AC electrokinetic technique that induces a net force onto dielectric particles in a non-uniform electric field [12]. As an active technique, DEP plays a very important role in trapping and separating particles at the micro scale. By varying parameters such as flow velocity, peak-to-peak voltage, phase, and frequency, particle separation can be achieved due to the difference of applied net force [13].

Traditional microfabrication techniques of microelectromechanical systems (MEMS) devices (e.g., lift-off and etching) have been employed to prepare micro-sized conductive patterns [14]. Although these techniques can provide a high-quality material and precise pattern, they are time-consuming, material wasting, use harmful chemicals, and require clean-room facilities such as photolithography equipment and vacuum metal deposition equipment [15]. Additionally, costly substrates such as silicon and glass wafers are usually employed for material deposition and microchannel construction. Hence, rapid prototyping is necessary for the design of microfluidic devices for quick testing of new configurations and functionality. In a rapid prototyping process, the microfluidic device can be fabricated directly from a digital design. 3D printing was used to construct a functional lab-on-chip device, which allows a researcher to design microfluidic devices for biological analysis without the skill of device fabrication [16].

Inkjet printing deposits material by creating droplets of ink which contain the material via a nozzle to the substrate. The use of inkjet printing technology offers a digital, contactless, and maskless process for material deposition. It has been considered as a promising technology for the additive fabrication of electronic devices [17]. A variety of materials have been prepared and printed, illustrating the capability of this technology. Inkjet-printing polymeric piezoelectric material was successfully prepared for micro-pumps [18]. This fabrication technique was also used to print conductive and adhesive material, constructing a wireless lab-on-chip device for the detection of a variety of fluids [19].

Metal is a good candidate for conductive electrodes, as its conductivity is much higher than that of conductive polymer [20]. Among them, silver is highly conductive and much cheaper than gold. In addition, silver nanoparticles are inkjet-printable [21]. Because of the nanoscale of metal particles, the sintering temperature of the ink could be very low, which makes it compatible with various substrates. Meanwhile, simple and cost-effective sintering methods have been developed for those conductive inks [22], such as chemical sintering [23], electrical sintering [24], plasma sintering [25], photonic sintering [26], and microwave sintering [21]. The printing of conductive patterns at micro-scale on polydimethylsiloxane (PDMS) for a DEP device was also investigated [27]. However, the utilisation of PDMS substrate caused major issues, such as cracking of the printed electrode during handling and heat treatment.

In this report, we present the utilisation of inkjet printing technology for the fabrication of microelectrodes in a practical application such as AC electrokinetic devices for microparticle manipulation. We investigated the possibility of printing a variety of electrode configurations. The heat treatment temperature is relatively low (ranging from 100 °C to 200 °C), and temperature was found to have a significant effect on the crystallinity and electrical resistivity of the printed material. Finally, a microfluidic device was successfully prepared and tested to demonstrate the workability of the proposed method.

The approach could be widely applied to prepare certain AC electrokinetic microfluidic chips for purposes such as the handling of microparticles in a continuous flow and other microfluidic device applications. Further improvements in the smoothness and precision of printed features might be executed using interpolation to incorporate the movement of the printer head in two axes.

## 2. Materials and Methods

**Figure 1:**
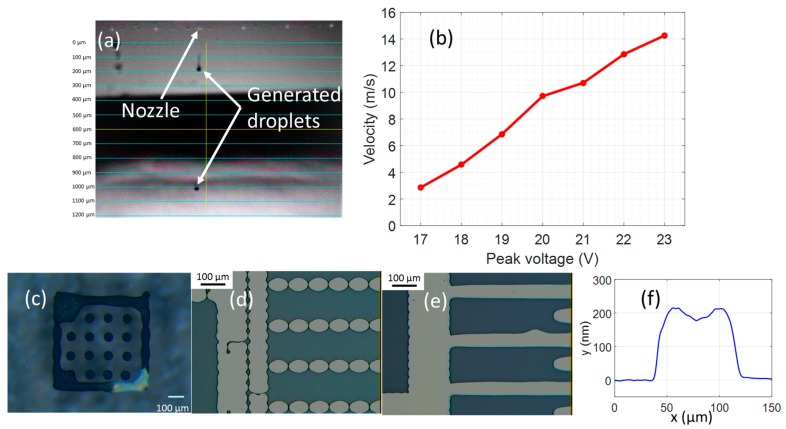
Printing process. (**a**) Photo of generated droplet at 20 V applied; (**b**) relationship of the velocity of generated droplets with the voltage applied to the piezoelectric-driven nozzle; (**c**) the spreading droplet array; (**d**) pattern printing with droplet space at 50 μm; (**e**) pattern printing with droplet space at 40 μm; (**f**) surface profile of the printed line.

A commercial Dimatix inkjet printer (DMP-3831, Fujifilm Dimatix, Inc., Santa Clara, CA, USA) was employed for all printing experiments. The printer was equipped with a standard 10 picoliter cartridge (DMC-11610) which has an orifice diameter of 21 μm. Each nozzle employs a piezoelectric element which is activated by an electric pulse to jet the ink out of the orifice in the form of droplets, as can be seen from Figure 1b. The waveform and peak voltage must be well adjusted in order to obtain stable droplet generation and velocity. Only one nozzle was employed to minimize the unexpected variation of droplet deposition. The pattern design was implemented using open-source software (i.e., Inkscape) and converted to a monochrome bitmap file using Microsoft Paint.

The glass cover substrate (size of 22 mm × 22 mm and thickness of 0.1 mm, Paul Marienfield, Lauda-Königshofen, Germany) was cleaned in acetone and rinsed with isopropanol to remove any organic residual before drying with an air blower. There was no plasma treatment applied to the substrate because the employed glass substrate is hydrophilic with the ink. During printing, the glass substrate was fixed onto the printer platen by the vacuum pump, which is embedded inside the printer.

We employed a commercial ink (Silver dispersion 736465, Sigma-Aldrich, St. Louis, MO, USA) which contains silver particles dispersed in triethylene glycol monomethyl ether (TGME) solvent. The nanoparticles had a diameter smaller than 50 nm. According to the supplier datasheet, the ink viscosity was 10–18 cP and surface tension was 35–40 dyn/cm. The density of the ink was 1.45 g/mL. These ink parameters are very important to the possibility of droplet generation.

During printing, the substrate temperature was set at 60 °C to enhance the vaporization of ink solvent. Hence, the spreading of ink to the substrate was eliminated, helping to make smaller features. In order to obtain stable droplet generation, cartridge temperature was selected to be 35 °C. Firing voltage was varied from 17 V to 23 V to observe the droplet velocity. For printing of the demonstration device, a peak voltage of 19 V was utilised. The resolution was set at 40 μm and 50 μm. Because the horizontal speed of the printing head during printing depends on the jetting frequency, a higher jetting frequency would result in a faster movement of the nozzles while jetting the nozzle. Hence, maximum jetting frequency was set at 2 kHz to reduce the horizontal velocity of the cartridge.

After printing, the substrate was kept in the printer platen for 10 min. Even with a heating temperature as low as 60 °C, the solvent of the deposited ink vaporized, and nanoparticles had partially sintered, appearing as a shiny surface. The printed pattern was then kept in an oven at different temperatures (i.e., 100 °C, 150 °C, and 200 °C) for 1 hour to enhance the sintering of the printed metal and improve the adhesion to the substrate. These heat treatments were conducted in ambient air.

Resistance of the printed material was measured using a digital multimeter (Agilent 34410A, Keysight Technologies, Santa Rosa, CA, USA). A scanning electron microscope (SEM, JEOL JSM 5600, JEOL Ltd., Akishima, Japan) was used to study the morphology of printed pattern in the micro-scale. Printed material crystallinity was studied using X-ray diffraction (XRD, Philips PW1820 Diffractometer, Philips, Almelo, The Netherlands). The profile was characterized with a profilometer (Form Talysurf PGI 2540, Taylor Hobson, Leicester, UK).

## 3. Results and Discussion

### 3.1. Droplet Formation and Printing

Figure 1a illustrates droplets that were stably generated from a nozzle when the activating pulse was applied. The image was captured after applying the pulse within a short period of 70 microseconds by the high-speed camera that is equipped with the printer. Figure 1b shows the relationship of applied voltage and velocity of the jetting droplet. A linear growth of velocity was observed when increasing applied activating voltage. At a peak voltage of 17 V, the velocity of the droplet was 2.86 m/s. It increased dramatically along with increasing voltage applied to the piezoelectric element. It reached 14.26 m/s under a driving voltage of 23 V. In this study, we used a peak voltage of 19 V to obtain a droplet velocity in the range of 6 m/s to 7 m/s.

The dimensionless grouping *Z*—which is determined by the ratio of Reynolds number to the square-root of the Weber number—is usually used to evaluate the inkjet printability of ink [28]:

(1) $$Z=\frac{Re}{{We}^{1/2}}=\frac{{\left(\gamma \rho a\right)}^{1/2}}{\eta }$$

where γ, η, and ρ are the ink physical properties, such as surface tension, viscosity, and density, respectively, and *a* is the diameter of the nozzle. From the ink specifications, the *Z* ratio can be derived with a value which ranges from 1.8 to 3.5, depending on the variation of surface tension and viscosity. It is commonly quoted that *Z* ratio must be larger than 1 and smaller than 10, although recent studies prove that inkjet printing can work in the assumed optimal range 1 < *Z* <14 [29]. Hence, the employed commercial ink is appropriate for inkjet printing.

The deposited droplets, surrounded by a square, are shown in Figure 1c, where a diameter of about 50 μm was commonly observed among the spreading droplet. However, discontinuous patterns were obtained if the droplet space was set at 50 μm, as can be seen in Figure 1d. When the droplet space was reduced to 40 μm, the overlapping of droplets was ensured, and a continuous pattern was obtained (Figure 1e). Hence, we employed the droplet space of 40 μm for all of the subsequent printed patterns.

Figure 1f shows the profile of a printed electrode on a glass substrate with a single layer of ink. The thickness of printed pattern is about 200 nm for single layer printing. It can be clearly seen that the printed pattern suffered from coffee-ring effect, where material focuses on the outer side of the printed profile, which makes the boundary become thicker than the centre area. The mechanism of this effect has been described as the result of the fast evaporation rate at the boundary of the droplet, which brings suspended material to the boundary of the pattern [30,31]. However, the depressed surface is minor compared to the thickness of the printed pattern. Hence, it should not have a significant effect on the device performance.

### 3.2. Morphology and Crystallinity

**Figure 2:**
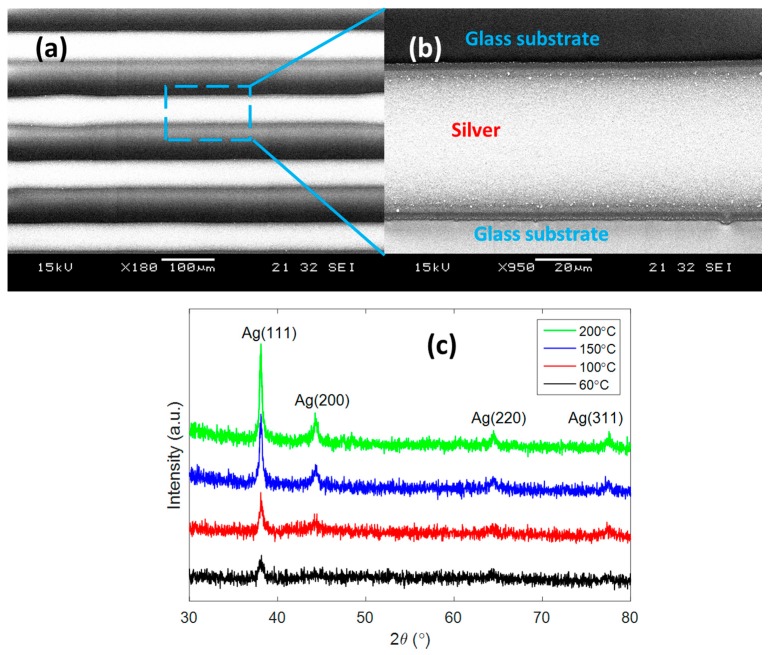
(**a**) Scanning electron microscope (SEM) image of printed electrodes; (**b**) surface of the printed electrode; (**c**) X-ray diffraction (XRD) pattern of printed film with different heat treatment temperatures.

The microscopic image in Figure 2a shows the printed silver after heat treatment of 150 °C with an array of lines which have a bright colour. It can be seen that the line boundary was not perfectly straight. It was observed that inkjet-printed patterns had a local bulge problem [27]. The enlarged image (Figure 2b) shows the printed pattern with a bumpy surface. This phenomenon could be the result of solvent vaporisation and the sintering of nanoparticles that form the rough surface. However, the printed line is continuous and crack-free thanks to the rigid glass substrate.

XRD patterns of printed films show a significant effect of heat treatment temperature on the crystallinity (Figure 2c). In all of the samples, four peaks at 38.1°, 44.3°, 64.4°, and 77.5° could be observed, which were indexed as (111), (200), (220), and (311) of face-centered cubic silver crystalline, respectively [32]. The peak (111) intensity increased from 33 to 187 when a sintering temperature of 200 °C was applied, which indicates that the heat treatment could improve the crystallinity of printed silver. In addition, the full width at half maximum (FWHM) value of the peak (111) reduced from 0.63° to 0.24°, which proves that the crystal size became larger as a result of the sintering. The XRD results prove that the silver nanoparticles were successfully printed, and patterns which have good crystallinity could be obtained by a low sintering temperature.

### 3.3. Electrical Property

**Figure 3:**
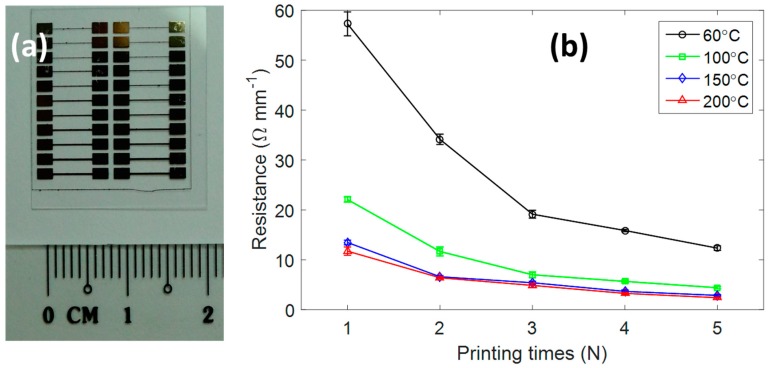
(**a**) Optical photo of printed line with different widths; (**b**) electrical resistance of printed lines with 5 mm length.

We investigated the effect of heat treatment temperature and the number of printing passes (*N*) on the resistivity of the silver pattern. The resistance of 5 mm-length line was measured as shown in Figure 3a. The as-printed pattern without heat treatment showed a high resistance of 57 Ω·mm^−1^ with a single printed line. It reduced following a nonlinear decay when increasing the number of printing passes. A line with five droplets printed overlap had a resistance of 12 Ω·mm^−1^.

When a heat treatment of 100 °C was applied, a significant decrease in resistance was observed. For example, the single printed line cured at 100 °C had a resistance of 22.1 Ω·mm^−1^. This value was cut down to 4.4 Ω·mm^−1^ when five printing passes was made. The increment of heat treatment temperature to 150 °C further reduced the resistance of the single line to 13.5 Ω·mm^−1^. However, 200 °C annealing could only make a slight improvement in the electrical resistance of the printed pattern (i.e., 11.7 Ω·mm^−1^). When treatment temperature increased from 150 °C to 200 °C, the resistance of the printed patterns did not notably change, and only slightly decreased.

The resistivity of printed lines could be calculated from their geometry (i.e., surface profile). The resistivity of the printed line was 1.62 × 10^−7^ Ω·m when the sintering condition was set at 150 °C for 1 h. The heat treatment at 200 °C could help to obtain the resistivity of 1.41 × 10^−7^ Ω·m, which is near the bulk silver resistivity of 1.59 × 10^−8^ Ω·m. It could be seen that the resistivity of the printed line was relatively small. In other words, the printed silver was highly conductive.

The well-crystallized material resulting from the heat treatment plays the main role in reducing the resistivity of printed material. It could be explained by the small size of silver nanoparticles helping to reduce the sintering temperature of nanoparticles. The as-printed silver with small-sized grains had a large grain boundary, which resisted the transport of free electrons in the metal network. The resistance reduced with the sintering effect because there is less grain boundary [33]. On the other hand, because it was observed that void space exists in printed silver nanoparticles, Krishnamraju et al. proposed that a higher sintering temperature could reduce the void space to improve the conductivity of the printed material [34].

### 3.4. Complex Shapes

**Figure 4:**
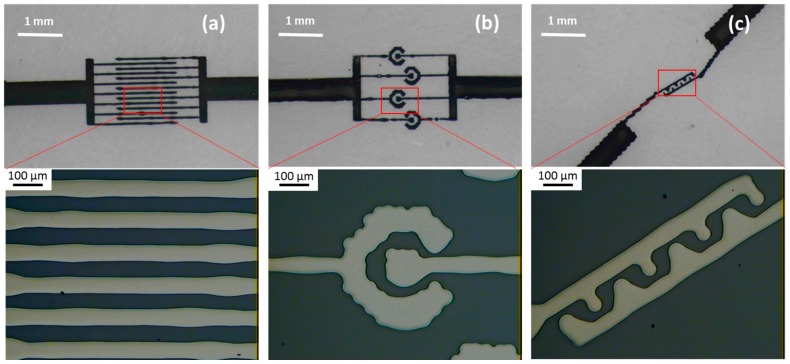
Printing various electrode designs. (**a**) Interdigitated comb-like electrode; (**b**) ring electrode; (**c**) castellated electrode.

The printing direction could significantly affect the smoothness of the printed pattern. The printer that we employed could only move in the *x*-axis in the horizontal plane during jetting of ink. Because of this movement during droplet travelling, the velocity of the droplet also contains a horizontal component. This horizontal velocity may cause the dislocation of a droplet when it touches the substrate. Hence, the best feature can be printed when the designed pattern is aligned with the printer’s moving direction. For example, a single line would be in the best shape if it was printed along the printer’s *x*-axis. In addition to printing a simple line, we conducted printing with different designs to demonstrate the flexibility of the fabrication method. We printed interdigitated comb-like electrodes with a feature size of about 60 μm by printing parallel lines with a single droplet in width, as can be seen in Figure 4a. The gap between two nearby electrodes was approximately 50 μm. Although the lines do appear to have a local bulge and the boundaries are not smooth, they are continuous and show no short-circuit between electrodes.

The ring electrode was printed as shown in Figure 4b. It can be seen that the feature boundary was not smooth due to the limitation in drop space and the size of the spreading droplet. Furthermore, the design contained a curving feature, while the printer could only perform a straight movement. Figure 4c demonstrates that the castellated electrode design was also printed. The main line was aligned along the movement direction of the printing head. The smallest feature size of about 60 μm is presented, and the printed pattern represents the design well. The smooth feature obtained suggests that a complicated configuration pattern could be printed with a printer that has interpolating movement in two axes; for example, performing movement in a curved trail as the design has specified.

### 3.5. Trapping of Micro Beads

**Figure 5:**
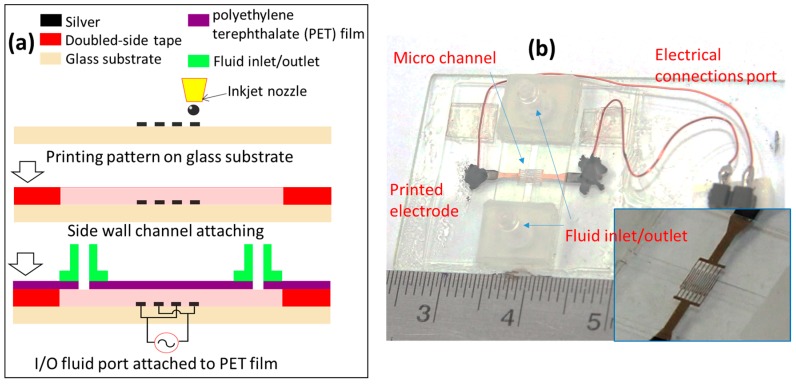
(**a**) Fabrication process of the dielectrophoresis (DEP) device; (**b**) optical image of the prepared micro-fluidic chip.

Printed comb-like interdigitated electrodes was used to demonstrate the workability of the method. A microfluidic device was prepared to demonstrate the utilisation of the printed electrode. The process to prepare a simple device is schematically described in Figure 5a. After printing and sintering electrodes, a double-sided tape with thickness of about 60 μm was cut to form a microchannel sidewall. Then, this double-sided tape was attached to the substrate as a spacer. A polyethylene terephthalate (PET) film covered the top layer as a lid, which formed a microchannel with a height of about 60 μm and width of 800 μm. The fluid inlet and outlet were bonded to the microchip using the double-sided tape. A silicone tube was used to connect the inlet with a syringe pump to supply the fluid flow. With low-temperature curing process, silver epoxy (Chemtronics, Kennesaw, GA, USA) was employed to bond the electrical wire with a printed pad to create the electrical connection without any damage to the printed pad. The silver epoxy included two parts which were mixed well before applying to the connect position. Because the cover glass substrate was too thin and easily broken, it was bonded to a glass slide as a rigid support to prevent cracking of the cover glass substrate. Figure 5b is an optical image of the prepared device with printed electrode, microchannel, and all electrical as well as fluidic connections. The inset is an enlarged image showing the microchannel.

Polystyrene beads with size 10 μm, mixed in water with the aid of a coupling agent, were purchased from Polysciences, Inc. (Warrington, PA, USA). The dispersion of beads was diluted with deionized (DI) water following the volume ratio of polystyrene dispersion:water of 1:5. The trapping of polystyrene particles was performed by applying an AC voltage to electrodes. Sine wave excitation voltage was supplied by a Function generator (Thurlby Thandar TG215, Thurlby Thandar Instruments, Huntingdon, UK). Fluid flow was generated by a syringe pump, which was set to deliver a constant flow rate of 4 µL/min. The applied frequency was set at 100 kHz, and the peak-to-peak voltage was *V*pp = 10 V. The optical microscope, equipped with a digital image acquiring system, was employed to capture the microparticles’ motion in the DEP device.

**Figure 6:**
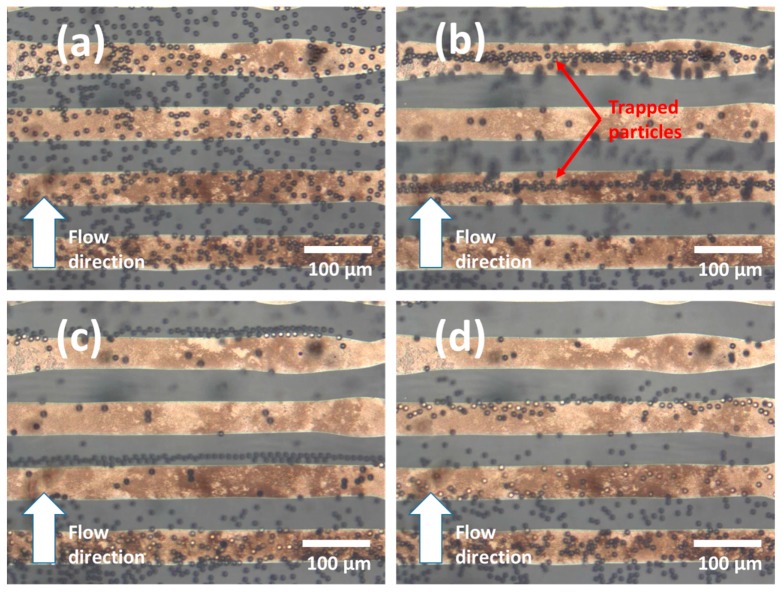
Comb-like DEP device for trapping electrode. (**a**) Randomly distributed 10 μm particle moving along with fluid flows in the microchannel; (**b**) when AC voltage was applied, the particles were trapped on top of the electrode; (**c**) when AC voltage was removed, the particle was released; (**d**) the particles return to move randomly.

Figure 6 illustrates the trapping function of the fabricated device. Without the applied voltage, polystyrene microparticles moved freely along fluidic flow due to hydrodynamic force, as shown in Figure 6a. The trapping phenomena immediately occurred upon the application of excitation voltage, where many particles were trapped along alternate electrodes (Figure 6b). It was observed that the microparticles were attracted to the centre of the electrode. We believe that the trapping force exerted upon the particles was a combination of two forces: DEP force and AC electro-osmosis force. When an AC voltage was applied, a non-uniform electric field was generated with high field strength at the electrode boundary. It is well-known that the suspension of beads in DI water will suffer positive DEP force at low frequency [27]. Due to the relative permittivity of polystyrene and DI water, positive DEP force occurred and tended to attract the particles to the electrode boundary. On the contrary, the AC electro-osmosis force brought particles to the centre of the electrode. AC electro-osmosis is a hydrodynamic phenomenon which creates rotating vortexes in the region near the surface of the electrode. The mechanism of this phenomenon is the motion of ions in the electrical double layer in the tangential electric field [35]. Meanwhile, the net force of DEP in the downward direction affected those particles in the middle of the electrode. The dominance of AC electro-osmosis results in microparticles being trapped at the centre of the electrode. However, it is noticed that some particles were not trapped at the electrode. It could be explained by the fact that the dominant trapping force—AC electro-osmosis—is only effective in the region near to the electrode. Therefore, the particles which were far from the electrode surface could pass through the trapping electrode.

Figure 6c shows the behaviour of particles right after the voltage was removed. Without the applied voltage, both DEP force and AC electro-osmosis would dissipate. Hence, the particles were released from the electrode and moved in the fluid flow due to the drag force, as can be seen in Figure 6d. The experimental results indicate that the printed electrodes are effective for application in trapping microparticles.

**Figure 7:**
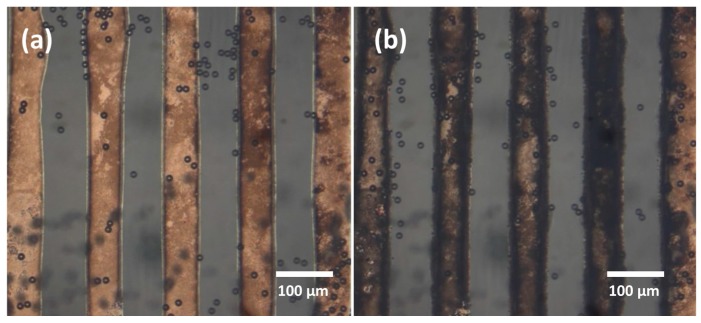
The corrosion of silver electrodes: (**a**) Electrodes before (**b**) and after applying AC voltage with peak-to-peak voltage of 14 V and frequency of 14 kHz.

However, the application of high voltage with low frequency caused severe damage to the printed electrodes, as shown in Figure 7. In Figure 7a, electrodes had a bright colour before turning the voltage to 14 V at the frequency of 14 kHz, at which point electrodes started to darken significantly (Figure 7b). At a low frequency, the effect of electrode damage was stronger. We believe that silver electrodes were corroded under the effect of applied voltage and the presence of water. The electrode functionality was lost, such that no trapping behaviour could be performed after the electrode darkening occurred. Hence, the damaging of silver electrodes would limit their application in low frequency and high peak-to-peak voltage.

## 4. Conclusions

We have employed inkjet printing as an alternative method to fabricate miniaturised electrodes for AC electrokinetics devices. The printing was performed by a commercial inkjet printer and silver nanoparticle ink using a cheap glass substrate. Continuous lines were obtained when a droplet space of 40 μm was set. Minimum feature size could be as small as 60 μm. Cracking of the printed pattern did not occur thanks to the utilisation of a rigid substrate. Heat treatment greatly affected the crystallinity of the printed silver. The printed material had low electrical resistivity after heat treatment at 200 °C (i.e., 1.41 × 10^−7^ Ω·m). In addition to a simple line pattern, some complex shapes were directly printed from the digital designs. The rapid prototyping and testing of AC electrokinetics devices on glass substrate were demonstrated using interdigitated comb-like electrodes. The functionality of the printed electrode (i.e., trapping of microparticles in a continuous flow) was performed successfully. The results show that inkjet printing is a powerful rapid prototyping tool for electrokinetic devices. The method could be applied to other electrode configurations, such as a 3D-structured electrode for more effective utilisation of the printed device.

## References

[1] Khoo Z.X., Teoh J.E.M., Liu Y., Chua C.K., Yang S., An J., Leong K.F., Yeong W.Y. (2015). 3D printing of smart materials: A review on recent progresses in 4D printing. Virtual Phys. Prototype.

[2] Salmoria G.V., Klauss P., Zepon K., Kanis L.A., Roesler C.R.M., Vieira L.F. (2012). Development of functionally-graded reservoir of PCL/PG by selective laser sintering for drug delivery devices. Virtual Phys. Prototype.

[3] Yee Y.W., Shyan J.Y.M. (2008). Hybrid approach in prototyping functional medical safety devices: A case study. Virtual Phys. Prototype.

[4] Saengchairat N., Tran T., Chua C.-K. (2016). A review: Additive manufacturing for active electronic components. Virtual Phys. Prototype.

[5] Van-Thai T., Yuefan W., Hongyi Y., Zhaoyao Z., Hejun D. (2017). All-inkjet-printed flexible ZnO micro photodetector for a wearable UV monitoring device. Nanotechnology.

[6] Zhang J., Yan S., Alici G., Nguyen N.-T., Di Carlo D., Li W. (2014). Real-time control of inertial focusing in microfluidics using dielectrophoresis (DEP). RSC Adv..

[7] Am J., Zhiwei Z., Kang Kug L., Chong H.A., Paul L.B. (2011). State-of-the-art lab chip sensors for environmental water monitoring. Meas. Sci. Technol..

[8] Dittrich P.S., Manz A. (2006). Lab-on-a-chip: Microfluidics in drug discovery. Nat. Rev. Drug Discov..

[9] Wang D., Zhu R., Zhou Z., Ye X. (2007). Controlled assembly of zinc oxide nanowires using dielectrophoresis. Appl. Phys. Lett..

[10] Nazmul I., Jie W. (2006). Microfluidic transport by AC electroosmosis. J. Phys. Conf. Ser..

[11] Walid Rezanoor M., Dutta P. (2016). Combined AC electroosmosis and dielectrophoresis for controlled rotation of microparticles. Biomicrofluidics.

[12] Pohl H.A., Crane J.S. (1971). Dielectrophoresis of cells. Biophys. J..

[13] Cui H.-H., Voldman J., He X.-F., Lim K.-M. (2009). Separation of particles by pulsed dielectrophoresis. Lab Chip.

[14] Li M., Li W.H., Zhang J., Alici G., Wen W. (2014). A review of microfabrication techniques and dielectrophoretic microdevices for particle manipulation and separation. J. Phys. D Appl. Phys..

[15] Vaezi M., Seitz H., Yang S. (2013). A review on 3D micro-additive manufacturing technologies. Int. J. Adv. Manuf. Technol..

[16] Ho C.M.B., Ng S.H., Li K.H.H., Yoon Y.-J. (2015). 3D printed microfluidics for biological applications. Lab Chip.

[17] De Gans B.J., Duineveld P.C., Schubert U.S. (2004). Inkjet printing of polymers: State of the art and future developments. Adv. Mater..

[18] Pabst O., Perelaer J., Beckert E., Schubert U.S., Eberhardt R., Tünnermann A. (2013). All inkjet-printed piezoelectric polymer actuators: Characterization and applications for micropumps in lab-on-a-chip systems. Organ. Electron..

[19] Cook B.S., Cooper J.R., Tentzeris M.M. (2013). An inkjet-printed microfluidic RFID-enabled platform for wireless lab-on-chip applications. IEEE Trans. Microw. Theory Tech..

[20] Hsien-Hsueh L., Kan-Sen C., Kuo-Cheng H. (2005). Inkjet printing of nanosized silver colloids. Nanotechnology.

[21] Perelaer J., de Gans B.J., Schubert U.S. (2006). Ink-jet printing and microwave sintering of conductive silver tracks. Adv. Mater..

[22] Wunscher S., Abbel R., Perelaer J., Schubert U.S. (2014). Progress of alternative sintering approaches of inkjet-printed metal inks and their application for manufacturing of flexible electronic devices. J. Mater. Chem. C.

[23] Grouchko M., Kamyshny A., Mihailescu C.F., Anghel D.F., Magdassi S. (2011). Conductive inks with a “built-in” mechanism that enables sintering at room temperature. ACS Nano.

[24] Mark L.A., Mikko A., Tomi M., Ari A., Kimmo O., Mika S., Heikki S. (2008). Electrical sintering of nanoparticle structures. Nanotechnology.

[25] Bromberg V., Ma S., Egitto F.D., Singler T.J. (2013). Highly conductive lines by plasma-induced conversion of inkjet-printed silver nitrate traces. J. Mater. Chem. C.

[26] Sowade E., Kang H., Mitra K.Y., Weiß O.J., Weber J., Baumann R.R. (2015). Roll-to-roll infrared (IR) drying and sintering of an inkjet-printed silver nanoparticle ink within 1 second. J. Mater. Chem. C.

[27] Kim Y., Ren X., Kim J.W., Noh H. (2014). Direct inkjet printing of micro-scale silver electrodes on polydimethylsiloxane (PDMS) microchip. J. Micromech. Microeng..

[28] Wang T., Derby B. (2005). Ink-jet printing and sintering of PZT. J. Am. Ceram. Soc..

[29] Torrisi F., Hasan T., Wu W., Sun Z., Lombardo A., Kulmala T.S., Hsieh G.-W., Jung S., Bonaccorso F., Paul P.J. (2012). Inkjet-printed graphene electronics. ACS Nano.

[30] Lim J.A., Lee W.H., Lee H.S., Lee J.H., Park Y.D., Cho K. (2008). Self-organization of ink-jet-printed triisopropylsilylethynyl pentacene via evaporation-induced flows in a drying droplet. Adv. Funct. Mater..

[31] Deegan R.D., Bakajin O., Dupont T.F., Huber G., Nagel S.R., Witten T.A. (1997). Capillary flow as the cause of ring stains from dried liquid drops. Nature.

[32] Kalimuthu K., Suresh Babu R., Venkataraman D., Bilal M., Gurunathan S. (2008). Biosynthesis of silver nanocrystals by bacillus licheniformis. Colloids Surf. B Biointerfaces.

[33] Shen W., Zhang X., Huang Q., Xu Q., Song W. (2014). Preparation of solid silver nanoparticles for inkjet printed flexible electronics with high conductivity. Nanoscale.

[34] Ankireddy K., Vunnam S., Kellar J., Cross W. (2013). Highly conductive short chain carboxylic acid encapsulated silver nanoparticle based inks for direct write technology applications. J. Mater. Chem. C.

[35] Oh J., Hart R., Capurro J., Noh H. (2009). Comprehensive analysis of particle motion under non-uniform AC electric fields in a microchannel. Lab Chip.

